# Dietary Modulation of Inflammation-Induced Colorectal Cancer through PPAR*γ*

**DOI:** 10.1155/2009/498352

**Published:** 2009-04-22

**Authors:** Ashlee B. Carter, Sarah A. Misyak, Raquel Hontecillas, Josep Bassaganya-Riera

**Affiliations:** Cell and Organism Section, Nutritional Immunology and Molecular Nutrition Laboratory, Virginia Bioinformatics Institute, Virginia Polytechnic Institute and State University, Blacksburg, VA 24061, USA

## Abstract

Mounting evidence suggests that the risk of developing colorectal cancer (CRC) is dramatically increased for patients with chronic inflammatory diseases. For instance, patients with Crohn's Disease (CD) or Ulcerative Colitis (UC) have a 12–20% increased risk for developing CRC. Preventive strategies utilizing nontoxic natural compounds that modulate immune responses could be successful in the suppression of inflammation-driven colorectal cancer in high-risk groups. The increase of peroxisome proliferator-activated receptor-*γ* (PPAR-*γ*) expression and its transcriptional activity has been identified as a target for anti-inflammatory efforts, and the suppression of inflammation-driven colon cancer. PPAR*γ* down-modulates inflammation and elicits antiproliferative and proapoptotic actions in epithelial cells. All of which may decrease the risk for inflammation-induced CRC. This review will focus on the use of orally active, naturally occurring chemopreventive approaches against inflammation-induced CRC that target PPAR*γ* and therefore down-modulate inflammation.

## 1. Introduction

The molecular basis for colorectal cancer (CRC) stems from genomic instability through genetic mutations linked to oxidative damage from chronic inflammation [1, 2]. Dissecting the inflammatory networks in the gut and identifying novel chemopreventive approaches are important because over 149 000 people will be diagnosed with CRC in 2009 in the United States; although there is a trend to a lower incidence that probably can be attributed to extensive screening and prevention efforts, almost 50 000 people will die of colon cancer this year [3, 4]. Furthermore, CRC is still the second leading cause of cancer-related mortality. The most common risk factors for CRC include genetic predispositions (adenomatous polyposis coli, hereditary nonpolyposis colon cancer) and exposure to radiation but intestinal inflammation (ulcerative colitis and Crohn's disease) also drastically increase the risk for developing colon cancer especially at early ages (<30 years of age) [5]. For instance, inflammatory bowel disease (IBD) and other types of chronic inflammation increase the risk for developing more severe colorectal cancer by 2 or 3 fold [2, 6]. Thus, the role of the immune system in the development and pathology of cancer is a large and growing area of research [1]. In addition, developing effective chemopreventive interventions is both timely and urgently needed.

The alimentary tract is a sterile organ at very early stages of development (i.e., embryonic and fetal phases). However, after birth, the gastrointestinal mucosa, particularly that lining the large intestine and terminal ileum, evolves to become densely colonized by bacteria. Specifically, from birth to weaning, successive waves of microorganisms will colonize the mucosa with a final result of 500–1000 species, which amount to 100 trillion discreet microorganisms, residing in the large intestine of adult humans [7]. The number of gut microorganisms is 10 times greater than the total number of somatic and stem cells [8]. In healthy individuals, these bacteria contribute to the regulation of T cell responses [9]. With a few exceptions, the lack of regulation leads to excessive polarization toward a T helper 1 (Th1) phenotype and initiation of IBD. The cellular interactions between T cells and antigen-presenting cells occurring in the gut mucosa and draining lymph nodes are tightly regulated to prevent excessive immune responses to foods and the gut microflora, whereas a defect in down-regulation of the immune responses predominates in individuals with IBD. The distribution and function of lamina proprial macrophages and T cells in the gut mucosa are important determinants of the extent and severity of the inflammatory process, and thus, represent targets for anti-inflammatory compounds. Bioactive food ingredients such as *n*-3 polyunsaturated fatty acids (PUFAs) and conjugated linoleic acid (CLA) are well recognized to suppress colonic inflammation and decrease the risk of colorectal cancer [10]. However, the limited understanding about their mechanisms of anti-inflammatory action has halted further progress in the development of nutrition-based approaches for preventing this devastating and widespread disease.

Many of the anti-inflammatory bioactive food ingredients and nutraceuticals elicit its actions through nuclear receptors, a broad class of transcription factors which regulate the expression of genes [11]. The peroxisome proliferator-activated receptors (PPARs), which make up a fatty acid binding subfamily of nuclear receptors, are involved regulating immune responses and therefore are potential therapeutic targets for CRC [12]. Nuclear receptors are common pharmaceutical targets for diabetes, IBD, endotoxemia. Of note, about 13% of all FDA-approved drugs target nuclear receptors [13]. Thus, they offer a great potential as targets for novel chemopreventive agents against both gut inflammation and inflammation-induced CRC.

## 2. Significance of CRC

Colorectal cancer is one of the most prevalent types of cancer, second only to lung cancer [14]. In 2004, over 35% of all men and women in the United States diagnosed with CRC died. The risk of colorectal cancer in the general population is less than 7%, though chronic inflammation including ulcerative colitis (UC) and Crohn's disease (CD)—the two clinical manifestations of IBD—can increase the risk of colorectal cancer. More specifically, the risk of developing colorectal cancer for an ulcerative colitis patient is estimated to be 2% after 10 years, 8% after 20 years, and 18% after 30 years of disease onset [15].

Demographics is another factor related to CRC, although not as strong as inflammation. Evidence suggests that ethnicity, gender, and geography all play a role. For instance, African American males over 50 living in the Western world are statistically at the highest risk for CRC [16]. Overall, inflammation emerges as the major factor for developing CRC. The consequences of CRC can be significant as the main therapies include colon removal which can lower dramatically a patient's quality of life, or lifelong surveillance including regular colonoscopies and medications, some of which have significant side effects [2]. In addition, in contrast to the more limited cost of chemoprevention, the cost of palliative treatment can represent a major burden for many CRC patients, with frequent hospital visits and expensive medications. Over 1.8 billion dollars are spent in medical care for IBD patients per year [16]. All of which could be minimized with more effective chemopreventive approaches.

## 3. The Role of the Mucosal Immune System in CRC

The mucosal immune system consists of cells and organs associated with body surfaces directly exposed to the environment, including lymphoid tissues associated with the lachrymal, salivary, gastrointestinal, respiratory and urogenital tracts as well as the lactating breasts [17]. The mucosal immune system is divided into inductive sites, where the immune responses are initiated or induced (antigen presentation to T cells) and effector sites, where responses are elicited (e.g., cytokine production, pathogen destruction).

Interestingly, about 70% of the immune system is localized in the gastrointestinal tract. Thus, understanding its architecture, functions, and how to favorable manipulate them is paramount for a rational design of chemopreventive approaches against both gastrointestinal inflammation and CRC. The gut associated lymphoid tissue (GALT) includes tonsils (lingual tonsils or Waldeyer's ring), adenoids (nasopharyngeal tonsils), appendix and specialized structures called Peyer's Patches, draining lymph nodes (i.e., mesenteric lymph nodes) in inductive sites and diffuse lymphoid tissue including intraepithelial lymphocytes and lamina propria cells (i.e., T cells, B cells, plasma cells, macrophages, dendritic cells, eosinophils, and mast cells) and isolated follicles in effector sites. Alterations in the GALT are well recognized to be an important mechanism underlying impaired host defense in CRC patients. Diet plays an important role in shaping the GALT as the lack of enteral delivery of nutrients decreases the numbers of T cells in the intraepithelial spaces and lamina propria of colorectal cancer patients [18]. In addition, the gastrointestinal tract is in very close proximity with the intraabdominal white adipose tissue (WAT) which in obese and overweight individuals is populated by large numbers of inflammatory macrophages [19]. Thus, the cross-talk between the GALT and WAT may play a role in shaping inflammatory responses that lead to CRC. In this regard, Crohn et al. in 1932 published a landmark paper characterizing for the first time Crohn's disease [20]. In this study, changes in the appearance in mesenteric adipose tissue were reported to be a key characteristic of the disease. In line with those early findings, we have recently found that macrophage infiltration into the intra-abdominal fat worsens the severity of experimental IBD [21]. Epidemiological evidence suggests a link between obesity and human CRC [22]. While the specific mechanisms underlying this link remain unknown, obesity-related inflammation represents a plausible explanation for this increased risk. The better understanding of the relationship between GALT and WAT may uncover some novel insights on mechanisms of carcinogensis and shed new light on possible preventive approaches.

The immune modulatory activities that aid in CRC chemoprevention by suppressing inflammation differ from the type of immune modulation after carcinogenesis has already occurred. Different modulatory agents should be sought for before and after carcinogenesis in order to heighten antitumor immune responses (i.e., T helper 1 and CD8^+^ T cell-mediated cytolytic responses) and to achieve optimal therapeutic efficacy. The mucosal immune system will play a crucial role in both phases of the disease. Interestingly, PPAR*γ* is expressed by all cell types that play a major role in the pathogenesis of CRC, including epithelial cells, T cells, and macrophages [23]. Therefore their function can theoretically be modulated by this nuclear receptor.

## 4. Inflammation-Induced CRC

It is estimated that approximately 15% of deaths in patients with CD and UC can be attributed to inflammation-induced CRC. The risk of developing CRC for CD and UC patients increases yearly, eventually reaching 12–20% increased risk after living with disease for 30 years [24]. Markers of inflammation like C reactive protein (CRP) whose synthesis occurs in hepatocytes and is induced by IL-6 and TNF-*α* in the serum have even been used as predictors of disease severity in advanced stages of CRC [16, 25]. While the exact mechanism for this elevated risk is still unknown, recent studies have been investigating whether the marked reduction in levels of the nuclear receptor PPAR*γ* in colons of UC patients may play a role in their increased susceptibility to developing colorectal cancer [26]. The exact mechanisms by which inflammation leads to CRC are slowly being elucidated.

The transcription factor nuclear factor kappa B (NF-*κ*B), which is found at the crossroads of many inflammatory pathways, has also been linked to tissue repair [27]. Aberrant NF-*κ*B signaling has been proposed to be one of the mechanisms by which chronic inflammation leads to cancer [28]. In a murine model of intestinal cancer, adenomatous polyposis coli (APC)^min/+^ mice, it was shown that the TLRs and IL-1R, through the adaptor MyD88, control the signaling of many genes which modify tumorigenesis in the intestine, including the NF-*κ*B-mediated genes IL-6 and IL-1*β* [29]. Loss-of-function studies demonstrated that MyD88-induced IL-6 was necessary for colon carcinogenesis [30].

Another upstream regulator of NF-*κ*B, tumor necrosis factor alpha (TNF-*α*), also plays a role in the development of IBD and CRC. TNF-*α* is a pro-inflammatory cytokine which activates NF-*κ*B as a positive autocrine feedback signal. Once activated, NF-*κ*B induces further production of TNF-*α* and other pro-inflammatory mediators [31]. The positive feedback loop between TNF-*α* and NF-*κ*B may lead to the overactivation of the NF-*κ*B tissue repair pathways, which in turn leads to tumorigenesis. Blockade of NF-*κ*B dramatically reduced tumor incidence by 75% in mice with DSS colitis [32]. Popivanova et al. more recently showed that treatment of DSS-challenged mice with a TNF-*α* inhibitor also reduces tumor incidence [33]. High levels of circulating TNF-*α* in plasma are associated with colorectal adenomas [34], further confirming the link between systemic inflammation and CRC.

One of the hallmarks of cancer cells is an uncontrolled growth and supported by a metabolic shift from aerobic to anaerobic metabolism [35]. This leads to an increased production of reactive oxygen species (ROS) in the electron transport chain. A state of chronic inflammation may also increase production of ROS as cytokines in inflammatory sites recruit macrophages and neutrophils which produce ROS. The ROS damage DNA which leads to mutations and the development of cancer [36]. ROS are also involved in pathways which progress tumor growth by increasing the production of interleukin 8 (IL-8) and inducible nitric oxide (iNOS), and inducing the secretion of matrix metalloprotease-1 (MMP-1) [37]. This illustrates an interaction between dysregulated host responses characterized by excessive inflammation resulting in genotype changes (i.e., mutations) that will in turn lead to CRC.

## 5. Role of PPAR*γ* in Regulating Inflammation-Induced CRC

The PPARs are a subfamily of nuclear hormone receptors which recognize a wide range of ligands, then heterodimerize with retinoid X receptor (RXR), and regulate expression of responsive genes. They also antagonize the activity of transcription factors, involved in inflammation and immunity such as NF-*κ*B, activator protein-1 (AP-1), nuclear factor of activated T cells (NFAT), and STATs [38]. Three different isotypes of PPARs, each of which represents a therapeutic target, have so far been identified: PPAR*α*, PPAR*δ*, and PPAR*γ* [39]. Of the three subtypes, PPAR*γ*, in particular, represents a potential therapeutic target for CRC and IBD chemoprevention. However, the apparent ability of PPAR*γ* to promote differentiation and maturation of epithelial cells has also led to studies of its potential role in the cause of CRC. Animal studies suggest both pro- and anticancer properties in the colon [40–42], though the bulk of studies point to PPAR*γ* ligands as chemopreventative agents [43].

PPAR*γ* is found in monocytes, macrophages, T cells, dendritic cells, skeletal muscle, adipocytes, and gastrointestinal epithelium and is involved in wide range of processes including the regulation of lipid and glucose homeostasis, inflammation, and adipocyte differentiation [38, 44]. One way by which PPAR*γ* acts in an anti-inflammatory capacity is through the inhibition of NF-*κ*B activity. More specifically, PPAR*γ* can interact directly with the NF-*κ*B subunits p50 and p65 [45]. In fact PPAR*γ* has been shown to antagonize the NF-*κ*B activities through several mechanisms. A nonpathogenic bacterium present in the human gut, *B. thetaiotaomicron*, acts in an anti-inflammatory capacity in Caco-2 cells by stimulating PPAR*γ* to act as a nuclear-cytoplasmic shuttle for the p65 subunit of NF-*κ*B. PPAR*γ* binds to the p65 subunit and prevents NF-*κ*B transcriptional regulation by exporting it from the nucleus [46]. In macrophages, sumoylation of PPAR*γ* ligand-binding domain prevents the removal of the nuclear receptor corepressor (NCoR)/histone deacetylase-3 (HDAC3) complex from the promoters of proinflammatory genes and, therefore, blocks NF-*κ*B [47, 48]. Stimulation of colonic epithelial cells with PPAR*γ* ligands prevents the immune-induced degradation of I*κ*B, anchoring NF-*κ*B in the cytosol [49]. Overall, the inhibition of NF-*κ*B in response to the activity of PPAR*γ* ligands attenuates the expression of various cytokines and inflammatory cells in colonic epithelial cells such as IL-1beta, COX-2, IL-6 IL-8, TNF-*α*, IFN-*γ*, and iNOS [48–51].

Increased expression of PPAR*γ* in human colorectal cancer cell lines treated with troglitazone, a PPAR*γ* agonist, is associated with increased differentiation [52]. This effect may exert itself due to the interaction of PPAR*γ* with the coactivator protein, Hic5, expressed in colonic epithelial cells. The expression of both Hic5 and PPAR*γ* is down-regulated in tumors. This interaction mediates induction of gut epithelial differentiation markers such as keratin 20 in gastrointestinal cells [53].

Pioglitazone, a synthetic agonist for PPAR*γ*, increases protein expression of caspase-3, a pro-apoptotic protein, and decreases protein expression of apoptotic inhibitory proteins Bcl-2, COX-2, and XIAP in retinoblastoma protein (RB)-deficient human CRC cells (SNU-C4 and SNU-C2A) [54]. These results have been demonstrated with several CRC cell lines using a number of PPAR*γ* agonists [55–58]. However, a limited number of mechanistic studies in inflammation-induced CRC are available using naturally occurring agonists of PPAR*γ*.

## 6. Potential Use of Naturally Occurring Agonists of PPAR*γ* for CRC Chemoprevention

A number of studies have examined the use of naturally occurring compounds for the prevention and treatment of inflammatory diseases and several types of cancer (Table 1). These naturally occurring compounds represent naturally occurring agonists of PPAR*γ* and/or potential therapies for inflammation induced-CRC. However, only a few have shown efficacy in the prevention or treatment of CRC. Among those are *n*-3 polyunsaturated fatty acids (PUFAs), which are known ligands or activators for PPARs. An active role for protein syndecan-1, which is regulated by PUFAs and a PPAR*γ* molecular target, has recently been identified in causing apoptosis in both prostate and breast cancer cells [83]. There is also some evidence to suggests docosahexaenoic acid (DHA), one of the PUFAs, is important in cellular apoptosis and cell cycle arrest in colorectal cancer. DHA and eicosapentaenoic acid (EPA) work at the molecular level through signaling pathways putting stress on proliferating cells and ultimately causing changes in gene expression of cancer cell lines [84]. DHA has generated promising results in animal studies with transplantable or chemically induced tumor. Success in some preclinical animal studies has led to clinical trials, many of which are ongoing, which use *n*-3 PUFAs as a nutritional supplement to reduce inflammation and modulate immune response [85]. Previously, we have demonstrated that dietary supplementation with conjugated linoleic acid (CLA) upregulated colonic expression of PPAR*γ* and downregulated colonic expression of TNF-*α* and inflammatory lesions in DSS-challenged pigs [86]. In DSS-challenged mice, immune and epithelial cell PPAR*γ* was required for the anti-inflammatory efficacy of CLA [75].

Another naturally occurring agent which shows promise in CRC is *γ*-Tocopherol—a vitamin that decreases COX-2 activity and nitric oxide (NO) expression [87]. The chemoprotective effect of *γ*-Tocopherol may also occur through an upregulation PPAR*γ* as treatment of human colon cancer cells (SW480 cells) with both 5 and 10 *μ*M concentrations of *γ*-Tocopherol resulted in an increase of both PPAR*γ* mRNA and protein expression [88].

The role of diet in colorectal cancer may be even more important than in other cancers due to the direct effect compounds have with the gut. This local effect has been shown to be critical for curcumin—a compound with negligible distribution outside the gut [89]. Broadly, methods of direct inhibition through nutraceuticals include reducing damage to DNA by neutralizing carcinogens, cytotoxicity or apoptosis of tumor cells, antiangiogenesis, and acting as anti-inflammatory agents [90]. In line with the concept of personalized medicine, some physicians recommend that people with a family history of cancers, or previously detected dysplasia or microtumors consume nutraceuticals first and foremost in the form of food and also in the form of multivitamins to decrease the risk of cancer [90]. However, the limited understanding of the mechanisms of action underlying the effects of botanicals, vitamins, and fatty acids has slowed down the rational development of preventive and therapeutic approaches against inflammation-induced CRC.

## 7. Conclusions and Future Directions

**Figure 1:**
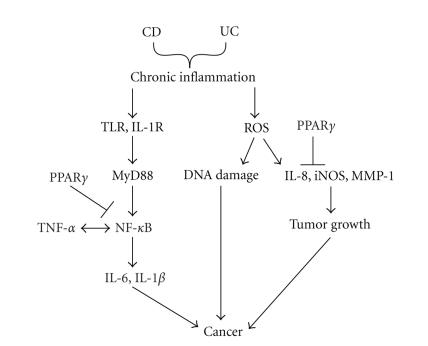
Chronic inflammation activates pathways leading to cancer. Chronic inflammation stemming from ulcerative colitis (UC) and Crohn's disease (CD), the two clinical manifestations of inflammatory bowel disease, activates nuclear factor-*κ*B (NF-*κ*B) downstream of MyD88 through the TLR and IL-1R. In turn, NF-*κ*B activation increases the expression of pro-inflammatory cytokines IL-6 and IL-1*β*. TNF-*α* also activates NF-*κ*B which, in turn, increases the expression of TNF-*α*, leading to a positive feedback loop between TNF-*α* and NF-*κ*B. Chronic inflammation also leads to the production of ROS which can damage DNA which can cause mutations responsible for tumorigenesis. ROS also cause the production of IL-8, iNOS, and MMP-1, which promote tumor growth. On the other hand, activation of PPAR*γ* can block carcinogenesis at two levels: (1) by antagonizing NF-*κ*B activity and (2) by suppressing IL-8 and iNOS expression.

People with CD or UC are predisposed to developing CRC, an outcome that depends on the extent and duration of chronic inflammation, which in turn is dependent on NF-*κ*B and ROS activity [6] (Figure 1). The key to maintaining homeostasis in the gut entails both downregulating inflammation as a chemopreventative method and promoting epithelial cell apoptosis and anti-tumor immune responses after the onset of cancer. As such, it is reasonable to suspect that introducing bioactive food ingredients which modulate NF-*κ*B or ROS pathways could be used to regulate inflammation before the onset of cancer. PPAR*γ* ligands have consistently acted as modulators and suppressors of NF-*κ*B activity [45–48, 91].

Thiazolidinediones (TZDs) are a well-known class of diabetes medication which acts as PPAR*γ* ligands. TZDs also suppress tumorigenesis in several types of cancers, including colon cancer [92]. Therefore, the identification of novel, naturally occurring PPAR*γ* ligands may represent a promising route of CRC chemoprevention.

There is also strong potential for the use of these bioactive compounds in conjunction with current treatments as this approach has already been shown to have positive results in cancer treatment [43].

The therapeutic effects of dietary CLA, and PUFAs through the activation of PPAR*γ* have already been summarized. Natural compounds for the treatment of CRC through PPAR*γ* have the potential to modulate the immune response and to be safe, easily accessible, and cost-effective. 

## Figures and Tables

**Table 1:** Natural compounds with therapeutic action against inflammation.

Compound name	Implications	Disease target	Study type	Molecular targets
Resveratrol (found in red wine, white hellebore) [59, 60]	Prevention	Skin cancer, colon cancer, diabetes, neurodegeneration	Cell culture, rodent, phase I clinical trial	Inhibition or reduction of COX-1 and COX-2, ROS

Beta-carotene (terpenoid found in yellow and orange fruits and vegetables) [61]	Prevention	Inflammation, high cholesterol	Mouse, ferret	ROS

Curcumin (spice derived from turmeric) [62, 63]	Prevention, therapy	Pancreatic cancer, myelodysplastic syndromes, colon cancer, psoriasis, inflammation	Cell culture, rat, clinical trials	Inhibits, COX activities, ROS, inhibits production of many pro-inflammatory cytokines (IL-8, MCP-1, TNF-*α*)

Folic acid (leafy vegetables and grain product) [64]	Prevention	Rectal health, pancreatic and CRCs	Cell culture/rodent	Involved in function, synthesis, and repair of DNA in cell cycle

Tocopherols (form of vitamin E found in oils and wheat germ) [65]	Treatment (supplement to anticancer drugs)	Prostate and lung cancers, CRC, and melanoma	Cell culture/rat	Mitochondrial interactions with compounds leading to apoptosis, upstream inhibition of NF-*κ*B

Omega-3 polyunsaturated fatty acids (PUFAs; found in grains, fish, and some oils) [66]	Treatment	IBD	Rat, mouse, human cancer cells	Cell cycle arrest and apoptosis through inhibition of the PI3-kinase signaling pathway, PPAR*γ* activation

Vitamin D or calcitrol (plant sources, fungi, dairy, and fish) [67, 68]	Treatment	Rheumatoid arthritis, dermatological conditions, osteoporosis, prostate, colon and breast cancers	Mouse, human clinical trial	Phase G0/G1 cell cycle arrest, regulation of cell cycle proteins, reduction of Akt and Erk which are cell survival markers

Calcium (dairy, nuts, seeds, soy, plants such as kelp and seaweed) [67]	Treatment/ prevention	Rheumatoid arthritis, dermatological conditions, osteoporosis, prostate, colon cancer, breast cancers and diabetes	Cell culture, mouse, human clinical trial	Balances cellular proliferation in the colon by inducing apoptosis

Dietary fiber (plant products, beans, root vegetables) [69, 70]	Prevention	IBD, CRC	Cell culture, human clinical trial	Butyrate, product of dietary fiber functions best with retenoids to inhibit histone deacetylase, butyrate regulates colonic epithelial homeostasis

Probiotics (fermented dairy) [71, 72]	Treatment/ prevention	IBD	Human clinical trial	Known mechanisms or targets are intestinal microflora, chemical balance, binds to carcinogens, production of short-chain FAs and anti-carcinogens

Prebiotics and synbiotics (oligosaccharides, garlic, onion, artichoke, and asparagus) [72, 73]	Treatment	Colitis, inflammation, cancer	Rats, clinical trial	Production of short-chain FAs, induce apoptosis of damaged cells, enhances activity of NK cells

Quercetin (a flavonoid found in cranberries and onions) [74]	Prevention	Breast, lung, skin, and colon cancers, heart disease	Cell culture, rodent	ROS, a proposed aryl-hydrocarbon receptor, suppresses pro-inflammatory mediators

CLA (dairy, meat) [75]	Prevention	IBD	Mouse, pigs	Enhances the immune function, activates PPAR*γ*, downregulates pro-inflammatory cytokines through NF-*κ*B

Ginseng (root of the panax plant in the family *araliaceae*) [76]	Prevention/ enhancement of current treatment	Colon cancer	Cell culture, mouse	Suppresses TNF-*α* and NF-*κ*B signaling decreasing inflammation. Also targets movement and angiogenesis of carcinoma cells

Turmeric extract (curry, curumin) [77]	Treatment	Arthritis, inflammation, cardiovascular disease	Rodent	Inflammation, angiogensis, eliminates free redicals and ROS, suppresses TNF-*α*

Phytoestrogen (soy products, whole grains) [78, 79]	Prevention	CRC, breast cancer, prostate cancer	Rat, clinical trial	Induction of apoptosis and inhibition of tyrosine kinases, regulates some pro-inflammatory cytokines (IL-6) hormones which induce cancer

Kaempferol (flavonoid in apples, onions, broccoli, and citrus fruits) [80]	Treatment/ prevention	Obesity and type II diabetes, cardiovascular diseases, neurodegenerative diseases, cancer	Cell culture	Upregulates TNF-related apoptosis-inducing ligand (TRAIL) receptors, sensitizes cancer cells to anti-carcinogenic compounds and pathways

Green tea phenols (tea leaves) [81]	Treatment	Gastric cancer and H. Pylori infections	Cell culture, mouse	Reduce ROS, gluthione metabolism, activates apoptotic markers and tightly regulates the cell-cycle

Blueberry Extracts (fruit from the shrub, *vaccinium cyanococcus*) [82]	Treatment	Cancer, inflammation	Cell culture, mouse	Inhibit growth, stimulate apoptosis
